# Dim Light at Night and Constant Darkness: Two Frequently Used Lighting Conditions That Jeopardize the Health and Well-being of Laboratory Rodents

**DOI:** 10.3389/fneur.2018.00609

**Published:** 2018-08-02

**Authors:** Mónica M. C. González

**Affiliations:** Sección Cronobiología y Sueño, Instituto Ferrero de Neurología y Sueño, Buenos Aires, Argentina

**Keywords:** lighting conditions, constant darkness, nightly dim light, laboratory rodents, circadian rhythms, suprachiasmatic nucleus, free running, health

## Abstract

The influence of light on mammalian physiology and behavior is due to the entrainment of circadian rhythms complemented with a direct modulation of light that would be unlikely an outcome of circadian system. In mammals, physiological and behavioral circadian rhythms are regulated by the suprachiasmatic nucleus (SCN) of the hypothalamus. This central control allows organisms to predict and anticipate environmental change, as well as to coordinate different rhythmic modalities within an individual. In adult mammals, direct retinal projections to the SCN are responsible for resetting and synchronizing physiological and behavioral rhythms to the light-dark (LD) cycle. Apart from its circadian effects, light also has direct effects on certain biological functions in such a way that the participation of the SCN would not be fundamental for this network. The objective of this review is to increase awareness, within the scientific community and commercial providers, of the fact that laboratory rodents can experience a number of adverse health and welfare outcomes attributed to commonly-used lighting conditions in animal facilities during routine husbandry and scientific procedures, widely considered as “environmentally friendly.” There is increasing evidence that exposure to dim light at night, as well as chronic constant darkness, challenges mammalian physiology and behavior resulting in disrupted circadian rhythms, neural death, a depressive-behavioral phenotype, cognitive impairment, and the deregulation of metabolic, physiological, and synaptic plasticity in both the short and long terms. The normal development and good health of laboratory rodents requires cyclical light entrainment, adapted to the solar cycle of day and night, with null light at night and safe illuminating qualities during the day. We therefore recommend increased awareness of the limited information available with regards to lighting conditions, and therefore that lighting protocols must be taken into consideration when designing experiments and duly highlighted in scientific papers. This practice will help to ensure the welfare of laboratory animals and increase the likelihood of producing reliable and reproducible results.

## Introduction

In mammals, light is sensed by three types of retinal cells (photoreceptors): the outer rods/cones and the inner ganglion cells. Through a visual pathway, rods and cones regulate vision and allow representation of the environment; rods are responsible for vision at low light levels (scotopic-night vision) while cones are active at higher light levels (photopic-day vision) and capable of color vision. In addition, based on the level of light intensity and schedule of exposure, light exerts profound effects on physiology and behavior. This “non-image forming” function of light contributes to the behavioral and physiological responses that occur to environmental lighting conditions via the hypothalamic suprachiasmatic nucleus (SCN), the brain's master circadian clock, and a direct pathway that is not supposed to include the circadian system (1–25).

Most physiological and behavioral functions of vertebrates exhibit a diurnal rhythm which is generated by the bilateral SCN. This rhythm exhibits self-sustained oscillations at regular intervals of ~24 h (circadian period; *circa diem:* near 1 day) in electrical activity, glucose utilization and gene expression (9, 11, 16, 26, 27). Its molecular machinery lies in individual pacemaker neurons, and their single-cell rhythmicity is driven by an intrinsic molecular transcription/translation oscillation loop in which protein products regulate the expression of “clock” genes (including the period genes *Per1* and *Per2*, and the crypotochrome genes, *Cry1* and *Cry2*) and the transcription factors *Clock* (Circadian Locomotor Output Cycles Kaput) and *Bmal1* (Brain and Muscle ARNT-like 1) (9, 28, 29). In addition to the SCN, there are central and peripheral tissues containing cells with self-sustaining circadian oscillations that control local time- and tissue-specific processes. These are known as “peripheral clocks” and include notably the olfactory bulb, neocortex, cerebellum, amygdala, caudate-putamen, nucleus accumbens, hippocampus, ventral tegmental area, dorsomedial hypothalamus (DMH) retina, heart, kidney, lung, gastrointestinal tract, and liver (4, 30–32). The timing (phase and period) of these peripheral clocks is controlled by the SCN in order to maintain coherence among a wide array of behavioral and physiological circadian rhythms; this process is known as “internal synchronization” (9, 11, 16, 27, 29, 30, 33, 34).

Gene activation-deactivation in the circadian master-clock is reset daily by external time cues called *zeitgebers* (“time givers”) that have the ability to adjust SCN clock mechanisms and overt circadian rhythms to environmental changes. The light-dark (LD) cycle is the most conspicuous *zeitgeber* for the circadian system and synchronizes the phase of endogenous rhythms with the appropriate daytime (light) and night (dark) phase; this process is known as “photoentrainment” (4, 7, 9, 14). The SCN predominantly entrains the circadian oscillations found in the hypothalamic–pituitary–adrenal (HPA) axis, the autonomic nervous system and neuronal systems to the LD cycle through the subparaventricular zone (SPV) and via relays in the DMH (15, 24, 27, 35–38).

In diurnal and nocturnal mammals, a subset of photosensitive inner retinal ganglion cells that express melanopsin (mRGCs) contribute to the non-visual response to light in collaboration with classical outer rods/cones photoreceptors. All these photoreceptors transform photic energy into an electrical signal (transduction) which passes to the brain in order to control circadian photoentrainment, locomotor activity, sleep, arousal, melatonin release, thermoregulation, heart rate, mood, cognitive functions, and pupillary light reflex (1, 4–7, 10, 12, 13, 17, 19–22, 25).

The retinal ganglion cells project to tens of brain regions and the mRGCS innervate most of them for integrating light responses and mediating photoentrainment of the circadian clock (3, 17, 18, 23). In agreement, the absence of mRGCs attenuates the phase resetting of light (1, 4, 10, 39–42). Photosensitivity is not uniform during the day. According to the time of day, light adjusts the spontaneous cycle of gene expression across the SCN to the day-night cycle, thus causing the phase shifts necessary for daily photoentrainment (2, 4, 9, 11, 16, 18). The most important time cues for resetting the principal circadian clock are the transitions between light and dark: light delays the phase of the endogenous rhythms over most of the night period while induces phase advances in the late night/early morning (4, 16, 43). For example, brief pulses of light at night have the ability to drop body temperature facilitating EEG sleep (“photosomnolence”) in mice (39, 44, 45). This drop in core temperature would itself modulate other responses to light mediated via mRGCs-SCN, ultimately resulting in phase shifts (44). Furthermore, an acute dark pulse given during the light period triggers an alerting response through mRGCs (39, 45). Indeed, when the period of SCN rhythms is shorter than the period of the environmental LD cycle, entrainment requires a small delay that can be accomplished by night light exposure. When the period of the SCN rhythm is longer than the period of the external light cycle, entrainment requires a phase advance that can be achieved by late night/early morning light exposure (2, 9). The resetting effect of light on peripheral clock expression and organ-specific output is thought to be transmitted via SCN control over hormonal pathways and autonomic innervation (46). For example, melatonin is a neurohormone synthesized and secreted by the pineal gland; secretion is facilitated during darkness and the circadian rhythm is maintained and entrained by a multi-synaptic SCN-sympathetic pathway. Pineal melatonin levels provide a direct entrainment of the peripheral clock together with SCN feedback, further synchronizing circadian physiology over the entire organism (47–49).

The SCN presents two coupled, but anatomically and functionally distinct, oscillators: the ventrolateral (VL) SCN sub-division or “core,” and the dorsomedial (DM) SCN, or “shell.” The VL SCN receives the major input from the retina via the retinohypothalamic tract; clockwork mechanism and dependent rhythms are entrained by light and oscillate with a period equal to the external LD cycle. The DM SCN is not entrained by light and runs free in a manner which is independent of the lighting conditions with dependent rhythms which also free-run and oscillate with a period equal to the master self-sustaining circadian oscillation (9, 27, 50, 51). The DM area requires the VL SCN to maintain circadian rhythmicity and keep the related rhythms in synchrony with the LD cycle (2, 27, 51–56). Coupling and synchronization between the molecular circadian oscillators is mediated by neurotransmitters (26, 57). At the molecular level, light-induced resetting is primarily mediated by the rapid induction of the clock genes, *mPer1* and *mPer2*, in the VL SCN, which then spreads to the DM SCN. In particular, the early night delay phase induces *mPer1* and *mPer2* initially in the VL SCN, which later induces *mPer2* expression in the rhythmic cells of the DM SCN. In contrast, the late night advancing phase is only accompanied by *mPer1* gene expression in the VL SCN, which then spreads to the DM SCN without significant induction of *mPer2* expression (2).

But how can SCN neurons manage to respond to light-dark transitions? The differing sensitivity of the retinal photopigments guarantees that the central clock will respond to irradiance and perceive the wavelength of transitions from twilight to daylight (blue-yellowish dark-light transitions) (58). The mRGCs are more sensitive to short wavelengths of light (blue light, 450–495 nm) than green light (495–570 nm) and are practically insensitive to long wavelengths of light (red light, 620–750 nm). Instead, in the majority of rodents, rhodopsin-rod cells transduce low light intensities and exhibit peak sensitivity at 498 nm (blue-green light), while cones are sensitive under brighter conditions and express cone opsins that are maximally-sensitive at 360 nm (UV) and 508 nm (green) light (58). The mRGCs receive inputs from both rods and cones and modulates their activity (17, 21). Rod-phototransduction converges into mRGCS through two pathways, the rod bipolar pathway for circadian photoentrainment at low light intensities, and the rod-cone pathway for circadian photoentrainment at high light intensities (1). Cones use a direct pathway to the mRGCs for photoentrainment (59). In direct response to light, the mRGCs become depolarized and via the release of glutamate from the retinohypothalamic nerve terminals, activate the SCN light-sensitive pacemaker neurons (25, 50, 51), although there are some differences in this mechanism across different species (60). Accordingly, blue light which represents the maximal sensitivity of the mRGCs, evokes the highest expression of *Fos, Per 1*, and *Per2* in the SCN (20).

By having their physiological and behavioral rhythms in phase, and coordinated with environmental lighting conditions, individuals have the capacity to make the homeostatic adjustments necessary for their adaptation throughout the day. The whole system is in harmony with environmental demands and is able to respond in a manner suited to a changing and predictable milieu. Light is the strongest environmental influence on circadian timing and altering the LD cycle from the solar cycle of day and night affects the integrity of the circadian system and the SCN-dependent rhythms start to run in a misaligned manner, both between each other and with respect to the LD cycle. This is known as “internal desynchronization” and has serious consequences for the brain and body in both the short and long term (11, 14, 34, 61–68).

Apart from the SCN, light may also regulate certain mammalian biological functions through circuits directly influenced by mRGCs, which include pretectal olivary nuclei, SPV, lateral geniculate nucleus of the thalamus (the ventral division and the intergeniculate leaflet), medial amygdala, lateral habenula, ventral preoptic area, and lateral hypothalamus. These are implicated in the effect of light on circadian entrainment and the modulation of sleep, mood, and cognitive functions and pupillary light reflex, crude vision, glare, heart rate, and cortisol levels (3, 4, 8, 13, 14, 18, 20, 21, 23, 69, 70).

It is assumed that experimental animals are raised and housed under strict and controlled standard lighting conditions as outlined in the *Guide for the Care and Use of Laboratory Animals*, which is published in a number of different countries. Nevertheless, certain lighting conditions, which have potentially long-lasting adverse outcomes on the welfare of laboratory animals, are often used in holding rooms and during tasks by animal care personnel and indeed, by some researchers.

The purpose of this review is to emphasize the need to avoid certain inadvertent or frequently used artificial lighting conditions on diurnal and nocturnal laboratory animals that challenge the appropriate development and function of the circadian system and other non-circadian brain areas. These artificial lighting conditions can impair the nervous system and alter physiological and behavioral processes in both the short and long term, thus invalidating experimental outcomes.

## Which lighting conditions should we be aware of? identifying the risks

Time of day and the wavelength/intensity of the photic stimulus are variables that we should take careful control of because of their capacity to directly affect biological functions and shift the master clock and/or modulate photic synchronization (20, 71). Even a brief light pulse at night elicits circadian rhythm phase shifts (18, 19, 71).

In mammals (including humans), the relevance of lighting conditions on health/disease begins as early as the intrauterine period, in which the circadian integrity of the mother is a key factor in conveying key environmental information related to time. During development, the fetal SCN is predominantly synchronized to the LD cycle by maternal hormonal signals and becomes vulnerable to environmental insults incurred by the mother (72–77). Once born, and until the SCN acquires its full functional capability (~3 weeks in rodents) (78–80), developing photoreceptors in the neonatal pineal organ, which will subsequently develop into typical secretory pinealocytes in adult (81), directly transmits information relating to environmental light conditions and collaborate with maternal cues in the regulation of biochemical rhythms during the first few days postnatally (79, 82). Thereafter, maternal entrainment declines and the development of a retina-SCN connection facilitates that this form of entrainment begins to be replaced by environmental cues such as the light-dark cycle for the photoentrainment of rhythms in the newborn (73, 74). As a direct consequence, the manipulation of gestational or postnatal photoperiods can influence the normal development and performance of the SCN, thus exerting influence over the outcome of circadian rhythms in both the short and long term (please refer to the following sections).

Most of us live under daily lighting schemes that do not follow the natural LD cycle in terms of schedule or the duration, wavelength and intensity of light, thus challenging our circadian system. In the evening, we are exposed for hours to incandescent bulb- and blue-enriched light from televisions, cell phones, and computer screens. Morning light has beneficial effects but at night can suppress melatonin concentrations, delay the onset of sleep by increasing alertness, decreases the efficiency of sleep at night and can reduce cognitive performance the next morning (8, 67, 83–90). According to the maximal sensitivity of the mRGCs, blue light evokes the maximal magnitude of the phase-shift for human alertness, thermophysiology and heart rate (6). Misalignment of our internal timing with socially imposed environmental lighting conditions (“social jetlag”) weakens synchronism between circadian rhythms and can exert dramatic effects upon physiology and behavior. Office workers and subway drivers that remain in dimly-lit environments for most of the day, along with frequent trans-meridian flyers and workers without a standard daytime schedule, are exposed to significant shifts in lighting cycles. As a result, irregular rhythms can emerge, which increase the risk of chronic health conditions such as sleep and mood disorders, cardiovascular and autoimmune diseases, cancer, obesity, stress, diabetes, premature aging, increased preterm births, spontaneous abortion, and lower birth weight (11, 63, 67, 68, 88, 89, 91–96).

Ordinarily, we would not even consider that such events could happen in animal housing facilities or experimental settings, since these animals must surely be held under safe and controlled lighting conditions. However, by lack of commitment or knowledge, the exposure of laboratory animals to aberrant lighting conditions is unexpectedly frequent.

### Lighting conditions in holding rooms

Since light represents the main environmental factor involved in the synchronization of biological rhythms and directly modulates sleep, mood and cognition, the consequences in terms of health and quality of life clearly depend on a regular LD cycle in agreement with local time, including sufficient light exposure time with adequate wavelength range and intensity during the day, and complete darkness at night. Based on scientific studies, worldwide guidelines for the management of environmental conditions recommend a semi-natural light cycle of 12:12 or 10:14 h with lighting within cages during the light phase below the threshold of aversion for mice/rats. For most pigmented strains, the recommended light level is below 60 lux, and for albino strains this is set to below 20–25 lux [(97)^1,2^, reviewed by (98, 99)].

Nevertheless, certain properties of light, such as intensity, wavelength range and distribution, are not clearly regulated by the Institutional Animal Care and Use Committee and are therefore frequently dismissed by providers and researchers. It is very common to find that rodents in holding rooms are exposed to different light intensities according to their location in the cage rack. Animals housed at the top of a rack are exposed, at the front of the cage, to more than 500 lux (up to 900 lux) while those at the bottom of the rack, nearest to the floor, can be exposed to 6 lux or less (97, 99)^1^, personal observation and personal communication with USA providers]. High light levels can dramatically affect retinal morphology and biochemistry. Consequently, rodents exposed to 130–270 lux above the light intensity under which they were raised are at risk of retinal damage (97, 102–104)^1^. A survey of Contract Research Organizations (CROs) located in North America, Europe, and Asia, using similar 12:12 lighting conditions, reported a high incidence of light-dependent retinopathy at a mean range of 210–490 lux in albinos (99). Susceptibility is likely to be greater for older animals (105), and in both young and old animals undergoing certain treatments that cause pupillary dilation (e.g., clonidine) (106) or exert direct effects on photoreceptors (107); this susceptibility can be dependent upon both strain (99, 105, 108, 109) and previous light-history (98, 109–113). Moreover, animals that are exposed for long periods to low lighting conditions are more susceptible to retinal damage in response to light (98)^1^. Thus, placing animals housed in the bottom of a rack on to shelves in the upper part of a rack is not recommended. Light-induced retinal damage is likely to cause differences in biological rhythm parameters between animals and thus lead to significant variation in experimental results. There is no information available (either published or via the manufacturers) relating to the light properties inside the newest ventilated cages; this represents an important gap in our knowledge.

To guarantee the accuracy and integrity of future research, laboratory animals should be kept under similar homogenous and safe lighting conditions during housing, transport and house-keeping tasks such as the cleaning and sanitization of cages and rack holders, in order to avoid changes in biological rhythms, which could, in turn, affect parameters associated with behavior and physiology (Boxes 1, 2).

Box 1 **Tips to measure light intensity**

- It is recommended to measure light levels using radiometric units based upon unweighted power measurements (e.g., μW/cm^2^) in place of lux measurements (100).
- Light intensity decreases with the square of the distance from its source (97) and light measurements depend on placement and orientation of the light meter in relation to the light source. Most cages for rodents have the food- and water-bottle holder as part of the lid, which systematically changes light intensity when compared to uncovered cages. To get reliable values, the actual light intensity checks must be performed with the light meter vertical to the light source at the animal's eye level while the cage is covered (with bottles and food), keeping the habitat intensity as imperturbable as possible during this procedure.
- Compared to clear plastic cages, tinted cages alter the photic environment and disrupt endocrine and metabolic circadian rhythms in albino rats (101). Thus, care must be taken when exchanging cages to maintain the same type of colored box. Therefore, transparency/color of the boxes must be indicated in Materials and Methods when publishing the results.

Box 2 **Recommendations**

- Cyclic light entrainment, is essential to maintain the robustness of the oscillators and circadian integrity.
- Animals must be under the regular support of an LD cycle with clear contrast between day and night, and with similar lighting conditions during the diurnal phase and complete darkness (0 lux) during the nocturnal phase.
- Light intensity should be monitored regularly within the cage at the animal's eye level to avoid experimental variability due to fluctuating lighting conditions.
- The lighting intensity inside of the home cage must be highly consistent between cages.
- The circadian system becomes sensitive to photic input at night. Thus, light pollution should be avoided, even low intensities of any color. To perform tasks, or during routine maintenance under darkness, use infrared goggles or maintain red-light intensity at 0 lux at the animal's eye level.
- The gestational or lactational photoperiods can shape behavior and physiology in the short- and long-term. Ask your provider for a report about the “lighting history” of your animals (LD cycle, intensity, and spectral transmittance) to confirm normal development and good health, and to adopt the appropriate habituation period to stabilize biological functions.

## Nocturnal dim light

In animal holding rooms, it is common to see light during the night through translucent observation windows and around doorframes as a result of the leakage of white light originating from constantly illuminated corridors. It is also frequent to expose animals to occasional dim red light during experimental procedures to facilitate tasks in the dark. Some researchers believe that it is possible to expose laboratory animals to low intensities of light similar to those experienced naturally by rodents at night (much like a moon-like glow, <1 lux) (114) and that this type of exposure is not harmless to health and will not cause functional changes in the circadian system. In contrast, light exposure during the night can have adverse effects on laboratory animals even at intensities below the thresholds required to stimulate light-responsive neurons in the SCN (0.1 lux in the rat and 1 lux in the hamster) and at wavelengths to which mRGCs are practically unresponsive (red light) (16, 115). However, phototransduction via the rods, which are the most sensitive of the photoreceptors, even at low light intensities, converges on mRGCs (4).

Dim illumination at night (“light pollution”) resets the circadian time-keeping system (115) of both the central and peripheral clocks, resulting in maladaptive or dysfunctional rhythms that increase the risk for chronic health conditions. Even a 1 h pulse of dim white light during the dark phase is enough to induce sleep in mice (19). Compared to the dark (0 lux), dim white light at night (DWL_N_) exerts both short- and long-term effects on the molecular clock, physiology, metabolism, hormonal rhythms, behavior, mood, hippocampal morphology, and cognition (Table 1). Dauchy et al. (130) described the detailed changes that can be made to a traditionally designed animal room in order to avoid light pollution, and how these improvements impact on the circadian regulation of biological rhythms.

**Table 1:** Summary of the main deleterious effects of dim white light at night vs. Dark (0 lux) on laboratory animal health.

	**Strain**	**Period of exposure-sex**	**Effects**	**References**
5lux		Prenatal Prenatal to P21 P21 ♂ ♀	↓ post-weaning growth rates In adulthood: ↑ anxiety-like behavior ↑ fearful responses to footshock	(116)
		P21 to adulthood P35 to adulthood ♂ ♀	In adulthood: ↑ anxiety-like behavior ↑ fear-like behavior ↑ fearful responses to footshock (under dim light since P35) ↑*clock* amplitude gene expression in hypothalamus ↓*Rev-ERB* gene expression in hypothalamus	(111)
	Mice (Swiss Webster)	P21 to adulthood ♂ ♀	In adulthood: -no change in body mass ↑ day-time food intake (females ate more than males) -no change in gonadal fat mass -no change in locomotor activity -no change in hepatic clock genes expression -does not affect glucose tolerance -no change in 24-h food consumption	(117)
		P35 to adulthood ♂ ♀	In adulthood: ↑ body mass (males) ↑ day-time food intake (males) ↑ gonadal fat mass (males) -no change in locomotor activity -no change in hepatic clock genes expression -does not affect glucose tolerance -no change in 24-h food consumption	(117)
		Adult ♂	↑ body mass ↑ day-time food intake -no change in locomotor activity -no change in plasmatic glucocorticoid concentrations ↓glucose tolerance -no change in 24-h food consumption	(118)
			-attenuated rhythmic expression *Per1, Per2, Cry1, Cry2* in SCN ↓PER1 and PER2 expression and attenuates rhythm in SCN ↓ rhythmic expression *Bmal 1, Per1, Per2, Cry1, Cry2* in liver ↓*Rev-erba* mRNA in white adipose tissue and liver during light phase -clock genes expression in hippocampus unchanged	(119)
			-overreaction of immune system	(120)
	Mice (C57bl/6)	Adult ♂	-anxiety-like phenotype - no change in circadian pattern of locomotor activity ↓ power of the locomotor activity rhythm	(121)
	Mice (C3H/HeNHsd)	Adult ♂	-depressive-like phenotype ↑ body mass ↓ hippocampal BDNF gene expression	(122)
		Adult ♂	-depressive-like phenotype -no change in locomotor activity ↓ dendritic length in dentate gyrus and CA1dendrites -impaired learning and memory	(123)
	Rat (Nile grass)	Adult (wild caught background) ♂	↑ immunological capabilities ↑ plasma corticosterone levels during active phase	(124)
5lux		Adult (ovariectomized)	-depressive-like phenotype ↓ density of CA1dendritic spines	(125)
	Siberian hamster		-depressive-like phenotype ↓ density of CA1dendritic spines ↓ locomotor activity during the night	(43)
		Adult (wild caught background) ♂	↓ post-saline (i.p.) locomotor activity in both dark and light phases ↓ immune function	(126)
	Rat (Wistar)	Adult ♂	↓ amplitude of the sleep-wake rhythm ↓ amplitude of the SWA and high frequencies in NREM sleep -induces an endogenous free run	(127)
0.11–1.08 lux	Syrian hamster	Adult ♂	↓ night-time pineal melatonin levels	(128)
0.2 lux	Rat (Sprague Dawley)	Adult ♂	-suppresses melatonin secretion -phase delayed glucose peaks -normoglycemic -phase-advanced lactic acid peaks -phase delay corticosterone peak ↑corticosterone levels -body growth unchanged	(129)

*BDNF, brain-derived neurotrophic factor; LD, light-dark cycle; P, postnatal day; SWA, slow-wave activity; SCN, suprachiasmatic nucleus; ZT, zeitgeber time*.

At 5 lux, DWL_N_ reduced post-weaning growth rates and had long-lasting effects by increasing anxiety-like and fear-like behavior and the fearful responses to foot-shock in adults that had been exposed pre-and/or post-natally (111, 116). According to the period of exposure during rearing, the metabolic functions during adulthood are affected differently by DWL_N_. Weanling mice, exposed since they were juveniles or adolescents, showed disrupted timing of food intake during adulthood by phase advancement of eating time and progressively increased daytime food intake; however, only males that had been exposed since adolescence showed increased body mass and gonadal fat mass relative to their dark counterparts in adulthood. During adulthood, none of these animals expressed alterations in glucose tolerance (117). In contrast to exposure at a younger age, exposure to DWL_N_ during adulthood reduced glucose tolerance and increased body mass; these are both factors that are indicative of a pre-diabetic-like state (118). More recently, Stenvers et al. (127) showed that in adults, DWL_N_ exposure reduced food intake and energy expenditure without affecting either body weight or impaired glucose tolerance. Certain differences in experimental procedures, such as the period of adaptation to DWL_N_, and the methodology used during testing, could be important factors for such discrepancy. These development-dependent alterations in metabolic energy homeostasis were associated with the disruption of circadian expression in crucial components of the molecular circadian clock in both the SCN and peripheral tissues that regulate metabolic functions. In particular, post-weaning exposure beginning at the juvenile or adolescent stage increases and decreases the amplitude of daily rhythm in the hypothalamic *Clock* and the expression of *Rev-ERB* in adulthood, respectively (111). However, during adulthood, DWL_N_ attenuates the rhythmic expression of the core circadian clock genes *Per1 Per2, Cry1*, and *Cry2* and PER1 and PER2 in the SCN, along with *Bmal1, Per1, Per2, Cry1, and Cry2* and *Rev-ERB* in the liver and adipose tissue (119).

Five lux of DWL_N_ also induces maladaptive behaviors such as reduced sleep efficiency, depression and cognitive impairment. The regulation of circadian sleep-wake rhythm, and synchronicity with the light-dark cycle, is achieved by both oscillators of the SCN via the SPV and mainly the DMH which conveys circadian information to both sleep and wake mechanisms (15, 24, 54, 131–133). DWL_N_ progressively reduces the amplitude of the sleep-wake circadian rhythm by gradually reducing sleep during the light (inactive) phase, but leaving the daily total sleep amount unchanged (127). These intrusions of waking episodes during the light phase reduce the amplitude of the daily rhythm of beta frequencies (16–19 Hz) and notably slow wave activity during non-rapid eye movement (non-REM) (127), both under strong influence from the endogenous circadian clock (134). It has been suggested that desynchronization between intra-SCN-oscillators was responsible for this circadian dysregulation, as shown by an endogenous free-running rhythm in locomotor activity with a period of ~25 h next to the entrained 24-h period (127). However, there is some disparity over this issue because investigators found no difference in the circadian pattern or daily locomotor homecage activity in mice (118, 121) or in diurnal rats (123). Moreover, depression is a persistent disorder that impairs mental and physical health. This condition has been historically associated with clock-related genes and dysfunction of circadian rhythms, particularly in terms of sleeping and waking (91, 135–138). Because of these alterations in the circadian system, adult nocturnal (43, 121, 122, 125), and diurnal rodents (123) under DWL_N_ have been shown to exhibit a depressive-behavioral phenotype. Under DWL_N_, adults also show reduced hippocampal expression of the brain-derived neurotrophic factor (BDNF) gene (122), a neuroprotective growth factor responsible for the growth and function of nerve cells and fundamental for plasticity, learning and memory. Hence, DWL_N_ make these animals more susceptible to stress-oxidative damage (139) and impairs spatial learning task performance and memory (123). Such changes in performance and memory are related to lower dendritic length in the dentate gyrus and the *Cornu Ammonis* 1 (CA1) in male diurnal rats (123), and a reduction in spine density (125) on the apical and basilar dendritic branches of CA1 pyramidal neurons in ovariectomized hamsters (43) which was not found in diurnal male rats (123). This discrepancy could be ascribed to the fact that ovariectomy *per se* causes a profound decrease in dendritic spine density in CA1 pyramidal cells of the hippocampus (135).

At 5 lux, DWL_N_ is also known to have a profound effect on the immune system in different species. When exposed to pathogens, diurnal rats (from a wild stock background) showed enhanced immune reactions that were associated with an increased concentration of plasma corticosterone during the active phase (124), while inbred laboratory mice over-reacted and showed elevations of the inflammatory response in the peripheral and central nervous system, along with sickness behavior (120). In addition, research has shown an impairment of immunological capabilities in hamsters derived from wild caught stock and held under an inverse LD cycle (126). It is possible that the disparity shown in previous studies could be attributed to certain critical aspects of methodology and differences in the resistance/susceptibility of the immune system between species. For example, laboratory mice show greatly reduced survival when exposed to the pathogen at the early evening (140). Moreover, delayed-type hypersensitivity (DTH) has been assessed during the rest period in hamsters and during the active period in diurnal rats; data indicate that the differential immunological outcomes could be related to daily DTH variation (124). Furthermore, hamsters and diurnal rats, when derived from wild-caught stock, may still retain some wild immunological traits (141) that differ from the antigen-inexperienced immune system of inbred laboratory mice.

Aside from illuminance, another property worthy of consideration is the wavelength of light at night. Certain wavelengths play a more relevant and efficient role and exert a greater perturbing effect on the circadian system than others. Green light exerts the maximum phase shift of locomotor activity (142), and blue light is the most efficient in suppressing pineal melatonin levels, followed by green light and yellow light, while near-ultraviolet and red light are the least efficient (143). More recently, Bedrosian et al. (43) conducted a study in ovariectomized hamsters that investigated the effect of different wavelengths of dim light (5 lux) on SCN-activation, mood, behavior and hippocampal cell proliferation. In line with previous studies, chronic DWL_N_ produces a depressive-behavioral phenotype (121–123, 125), although mood disorder was considerably more pronounced in animals under dim blue light at night (DBL_N_) (43). Moreover, like DWL_N_ (43, 125), DBL_N_ reduced the number of dendritic arbors in the CA1 region of ovariectomized hamsters (43). Furthermore, unlike juvenile and adolescent mice (117), ovariectomized hamsters exposed to DWL_N_ expressed reduced locomotor activity in holding cages during the dark phase with 24 h lower power than a nightly dark control group, while dim red light (DRL_N_) resulted in an increase of activity. DBL_N_ did not significantly affect total activity but induced the greatest reduction in the 24 h power of activity rhythm (43). Moreover, during the early subjective night, a 5 lux brief pulse of DWL_N_, and notably of DBL_N_, activated the retinorecipient-SCN region, in a manner similar to a 150-lux pulse of white light (43). In agreement with this finding, a pulse of blue light (250 lux) during the dark phase evoked the greatest increase of *Fos, Per1*, and *Per2* expression in the SCN and the adrenal gland, resulting in the highest levels of corticosterone secretion, which are normally associated with behavioral arousal (20). It is worth noting that while 250 lux blue light evokes higher gene induction in both the SCN and adrenal gland resulting in higher corticosterone levels and wake, the same intensity of green light produced a greater response in the major sleep promoting area (the ventrolateral preoptic area, VLPO), lower responses in the adrenal gland and corticosterone levels, and induces sleep. This opposite wavelength effect is melanopsin-dependent and provided evidence of two potentially different pathways with different spectral sensitivities for the wakefulness and sleep-promoting induction of light in nocturnal rodents (20).

To assess the effect of continuous dim light at night, all of the studies mentioned above used 5 lux night-time light, which is five times brighter than maximal moonlight (<1 lux). However, it is important to highlight the fact that lower intensities of light can also affect the circadian timing system.

DWL_N_ of low light intensity (0.2 lux; 0.08 μW/cm^2^) disrupts the normal circadian output from the peripheral clocks (144). Indeed, peaks in glucose plasma concentration were phase-delayed by 4 h although animals remained normoglycemic throughout the entire 24-h period. Plasma lactic acid concentration remained unchanged, although peak levels were phase-advanced by 4 h, thus increasing its level of uptake during the night; corticosterone showed elevated daily plasma levels with peak amplitude delayed by 4 h. Plasma levels, and the diurnal rhythm of total fatty acid and lipid fractions, along with dietary and water intake and body growth rate, were similar to a 12:12 LD cycle without light pollution (129). Light is a key factor that controls the night-time secretion of melatonin from the pineal gland (144) and at 0.20 or 1.08 lux, DWL_N_ suppresses the levels of melatonin in both the plasma (129) and pineal gland (128) of rats and hamsters, respectively, in a manner similar to nightly white bright light (300 lux) (129). As melatonin restricts tumor growth (145), rats exposed to 0.20 lux DWL_N_ also showed an enhanced tumor growth rate (146, 147). Even low intensity green light (0.005 lux) may entrain the activity rhythms of hamsters exposed to different entrainment paradigms (148).

In short, animals must be protected from nightly light exposure at any intensity and wavelength. The studies described herein clearly highlight that there is a clear connection between dim light at night and maladaptive behavioral and physiological outcome with both short- and long-term effects. Compared to the dark (0 lux), light spectral transmittance associated with a blue wave-length (white light and notably, blue light) has the most perturbing effect upon the circadian system, behavior, synaptic plasticity, metabolic function, immune system, mood and cognition, even at very low intensities. These behavioral and physiological consequences would be consistent with the coding of different wavelength sensitivities attributed to each type of photoreceptor, and the predominant participation of melanopsin-RGCs (which are particularly sensitive to blue light) in order to convey the stimulus to circadian and non-circadian systems.

## Constant darkness

The synchronization of behavioral and physiological circadian rhythms can only be achieved through the entrainment of both SCN oscillators by a time signal environment with circadian characteristics close to the solar LD cycle. In the absence of light, and without other external time cues, the period of the circadian rhythms can be controlled by the intrinsic oscillation of their specific central clock (referred to as “*free running rhythm*”) (9, 27); these rhythms then begin to free run with different period lengths and a lower amplitude (34, 149). This tool is used by chronobiologists to eliminate entrainment effects by light and therefore “unmask” the endogenous rhythm created by the machinery involved in the specific central clock.

Despite the elevated risk of depression in response to limited light exposure/intensity (138, 150), the consequences of long-term total darkness (*free-running* conditions) on behavior and neuronal systems were only reported a few years ago in laboratory animals (151, 152). For example, rats under complete darkness (0 lux) for several weeks exhibit distinct behavioral and anatomical features that are characteristic of depressed patients (Table 2). Behaviorally, these animals exhibited a delayed sleep phase and a lower amplitude sleep-wake circadian rhythm which was caused by an increase in sleep during the active period (151); these animals also showed increased immobility in a modified forced swim test (FST), increased locomotor activity in a novel environment, and a sensitized response to subsequent stressors (152). Anatomically, these animals expressed increased levels of apoptosis in the three monoaminergic systems associated with the pathology of depression, namely the noradrenergic (NA)-locus coeruleus (LC), the serotoninergic-raphe and the dopaminergic-ventral tegmental area systems, as well as a significant and continuous reduction in the number of NA boutons in the frontal and prefrontal cortex (151, 152). Long-term light deprivation (DD) did not affect body weight gain or adrenal weight, indicating that it is not stressful and therefore that damage to the NA-LC was not the result of chronic stress (152)]. In this study, apoptotic death was progressive but could be restrained by the chronic administration of the tricyclic antidepressant desipramine, which also causes NA axonal regrowth and amelioration of the depressive behavioral phenotype, thus validating light-deprived rats as a non-stressful and genetically-intact model of depression (152). The depressive profile induced by light deprivation is not an acute effect, since an extension of 2 h to the dark period of LD animals (12:14) for 1 day did not affect immobility time during FST during the last hour of darkness (152). These results suggested that the absence of light input to brain centers which control mood disorders may be involved in the etiology of depression. As for the neural component, we originally proposed that the absence of light input would interrupt the synchronism between the SCN oscillators and dissociate rhythmic outputs, thus leading to depression after the dysregulation of the transynaptic efferent circuits that modulate the brain's monoaminergic systems and their associated behaviors (sleep, arousal, motivation, and mood) (151, 152). In agreement, more recently, we showed that a depressive phenotype can emerge from the desynchronization of cellular oscillators within the master circadian clock itself (164), suggesting for the first time in a genetically and neurologically intact animal, that the biological clock can exert functional implications upon the etiology of mood disorders and the specific mechanisms which underlie the beneficial effects of bright light therapy. Certainly, an impact of DD on the direct photic regulation on sleep and mood cannot be ruled out.

**Table 2:** Summary of the main deleterious effects of constant darkness (0 lux) on laboratory animal health.

**Strain**	**Period of exposure—sex**	**Effects**	**References**
Rat (Sprague Dawley)	Adult ♂	- delayed sleep phase ↓ amplitude of the sleep-wake rhythm ↓ NA fibers and boutons in Frontal Cortex	(151)
		- depressive-behavioral phenotype - sensitized response to acute stress ↑ locomotor activity in a novel environment - neuronal death in the NA-LC, 5-HT-DRN, DA- VTA ↓ NA fibers and boutons in Prefrontal Cortex	(152)
		↑ Pyroglutamyl-peptidase I in retina and anterior hypothalamus	(153)
Rat (Wistar)	Prenatal-P49 ♂♀ tested in adulthood	↓ neurogenesis in dentate gyrus (lower in ♂) -spatial memory impairment (worst in ♂)	(154)
	P0–P24 ♂♀ tested the following 3 days under a LD cycle	↓ number of neurons and glial cells in the SCN	(155)
	Adult ♂	-depressive behavioral phenotype	(156)
		↓ body weight gain ↓ c-Fos positive cells in the SCN	(157)
Mice (C57BL/6J)	P0–P21 ♂♀ followed by 24 h LD cycle • tested at P1–P50	↓ dam body weight during suckling time -no change in maternal behavior or stress markers At P10: ↓ body weight ↑ glucocorticoid receptor protein expression at ZT16 in the PVN	(112)
	P0–P21 ♂♀ followed by 24 h LD cycle tested at P50	-depressive-like phenotype ↓ glucocorticoid receptor in dentate gyrus, no changes in PVN ↑ plasma corticosterone level at ZT12 (♂) in response to acute stress - no change in *Crh*mRNA in the PVN	(158)
	Adult ♂	- depressive-behavioral phenotype ↑ levels of IL-6, IL-6 mRNA, NF-κB p65, phospho-NF-κB p65, and phospho-IκBα in hippocampus	(159)
Mice (ICR)		-depressive-behavioral phenotype (more severe in ♀) ↓ intrinsic excitability of layer V pyramidal cells in motor cortex (lower in ♀)	(160)
	P0–P21 ♂♀ tested at P22–P35	- depressive-behavioral phenotype - weakened locomotor activity - layer V pyramidal cells in motor cortex ↓ morphological complexity ↓ intrinsic excitability ↓ burst-firing	(161)
Mice (YFP-H)	P0–P21 ♂♀ tested at P22-P35	↑ dendritic arbors density in RGCs (subtype RG_A_) ↓ morphological complexity of layer V pyramidal cells of both primary visual and auditory cortex	(162)
Mice (C57BL/6N)	Adult ♂	- depressive behavioral phenotype ↑ IL-6 plasma In hippocampus: ↓ cell proliferation ↓ per2 protein level ↑ npas2 protein level ↑ IL-6 and IL-I receptors protein levels ↑ NF-κB DNA binding activity	(163)

*BDNF, brain-derived neurotrophic factor; DA, dopaminergic; VTA, ventral tegmental area; LD, light-dark cycle; NA, noradrenergic; LC, locus coeruleus; P, postnatal day; RGCs, retinal ganglion cells; serotoninergic (5-HT)-dorsal raphe nucleus; SWA, slow-wave activity; SCN, suprachiasmatic nucleus, ZT, zeitgeber time*.

Subsequently, several studies have confirmed our results and additionally reported a range of other deleterious effects associated with DD. For example, light-deprived rodents expressed a depression-like phenotype (112, 156–161, 163), with more severe symptoms evident in females than males (160). Furthermore, in light-deprived adults, the enzymatic activity of pyroglutamyl-peptidase I in the retina, and anterior hypothalamus, was increased, thus indicating dysregulation in the metabolism of neuropeptides in the nervous system including alterations in reproductive function (153). The absence of photic stimuli also reduced neuronal activation in the SCN without affecting the circadian rhythmicity of corticosterone and melatonin plasma levels and increased and decreased body weight gain after 3 and 7 weeks, respectively, without affecting daily food ingestion (157). However, we did not find any differences in body weight gain after 3–6 weeks in DD rats (152). We should also mention that these authors have previously submitted animals to an inverted LD cycle and weighed them after behavioral stressful tests (by their characteristic overreaction to stress, the body weight of these animals may also have been affected), while our DD animals were previously housed in a 12:12 LD cycle and weighed before the behavioral test.

Apoptosis, along with neurodegeneration and inflammatory diseases, is associated with an accumulation of oxidative damage caused by the overproduction of free radicals such as reactive oxygen species (ROS) (165, 166). Accumulative oxidative stress reduces the levels of BDNF (167), a neurotropin that promotes neuronal survival, and its synaptic plasticity effectors, synapsin I and cAMP response element binding protein (CREB) (167, 168). Increased oxidative stress and reduced BDNF expression and/or function, particularly in the hippocampus, have also been implicated in both the pathophysiology of major depression and the typical overreaction to stress (169, 170). Confirming light-deprived animals as a model of depression, DD was shown to aggravate the outcome of oxidative stress, as shown by proinflammatory processes in the hippocampus (159, 163) which impaired neural plasticity and neurogenesis resulting in a subsequent deficit of learning (154), susceptibility to stress (152), and reduced c-Fos and CREB expression in several limbic areas involved in the reward and motivation mechanism (171). The depressive behavioral phenotype, inflammatory profile, and associated hippocampal neurodegeneration correlated with the DNA methylation–related chromatin remodeling process (172) that regulates neuronal activity dependent of BDNF gene expression (173). It was suggested that the circadian system would contribute to cellular homeostasis by regulating the expression of neuroprotective proteins that curtail oxidative damage and prevent synaptic damage in the nervous system (174). In agreement with this, transgenic mice featuring the knockout of the brain-specific clock genes *Bmal1* or *Clock* and neuronal PAS domain protein 2 (*Npas2*) show severe astrogliosis, oxidative damage and exacerbated neurodegeneration (175), thus suggesting that alterations in the hippocampal protein levels of the clock genes *Per2* and *Npas2* induced by DD (163) would be involved in neuronal death, and the morphological and synaptic changes described previously. This action would be facilitated by increased binding activity of NF-κB DNA (163), a transcription factor that is activated by ROS and regulates the inflammatory and immune response (165). Moreover, Qi et al. (176) recently found that the apoptosis triggered by DD extends to the liver due to the oxidative damage caused by the overproduction of ROS after a marked reduction in the mRNA levels of antioxidant enzymes.

The harmful effects of DD occur relatively swiftly and cannot be rapidly undone. In a previous study, 7 days were enough to elicit a depressive behavioral phenotype that could not be reversed by 1–2 days in 12:12LD (156). This time period was also sufficient to reduce levels of BDNF mRNA and protein expression in the visual cortex and affect the regulation of hippocampal genes that are critical for neural plasticity (172).

The effects of the absence of light when the SCN is still immature and has not yet completely configured its synaptic connections (78, 80) are reflected by long-term consequences on circadian rhythm outputs, clock gene expression, mood, physiology, stress markers, neuronal function, plasticity, and cognition. During the suckling period, DD causes a reduction in the number of neuronal and glia cells responsible for the coupling between SCN oscillators (155). This structural alteration will certainly affect the development and performance of the SCN, which may, in turn, desynchronize rhythms and modify animal physiology and behavior. Restricted to certain developmental periods, light deprivation causes morphological and physiological ontogenetic changes when compared against normal maturational processes, which may result in a depressive phenotype in juveniles and into adulthood, together with other benchmarks of clinical depression which are not prevented by post-weaning housing under a 12:12 LD cycle (158, 160, 161). In particular, pups raised up to the weaning stage in DD, followed by standard 12:12 LD conditions, showed a depressive-like phenotype during adulthood, with reduced expression of the glucocorticoid receptor in the hippocampus but without changes in the paraventricular nucleus (PVN). These effects were seen with higher plasma corticosterone levels in response to acute stress only in males, indicating sexual dimorphic sensitivity in the nervous system during development to the lighting conditions (158). Only at 10 days of age (P10), did pups raised in DD show reduced body weight and increased expression of glucocorticoid receptor mRNA in the paraventricular nucleus (PVN), thus leading to a higher level of activity in the hypothalamic-pituitary-adrenal axis (112). Light deprivation did not alter the levels of maternal stress markers and did not change maternal care behavior (except for a different temporal distribution) but did reduce body weight in dams during the suckling stage (112). This result ruled out the fact that altered maternal behavior may have been a factor responsible for the changes found in adults reared under DD and also highlight the absence of light as a main trigger that would affect the HPA axis only at a specific development stage. The absence of photic stimuli during the suckling period also reduced the morphological complexity of the primary visual and auditory cortical layer V pyramidal cells, with increased morphological complexity in a specific mRGC subtype (mRGC_A_ cells), as shown later during the pre- and peri-puberal periods, suggesting that postnatal DD promotes “cross-modal plasticity” among different neuronal systems during brain development (162). Moreover, light-deprived animals from the prenatal period to adulthood showed reduced levels of neurogenesis in the dentate gyrus, which was more severe in adult males than females and associated with subsequent impairment of spatial memory (154).

It was suggested that the dimorphic behavioral-depressive phenotype described in previously reared-DD juveniles was associated to weaker locomotor activity during stressful tests (161) and that this was related to the simplified morphological complexity and lower excitability of the motor cortical dendritic arbors of layer V pyramidal cells (the major output from the cortex) exhibited in cortical slices, principally from females (160, 161). We have previously shown that in adult rats under DD, the increased immobility during FST was not caused by a deficit in motor activity or a response to salient stimuli. Indeed, these animals showed increased ambulatory activity in a novel environment (152). Moreover, and contrary to the findings of Lu et al. (160) and Zhang et al. (161) light deprivation did not affect motor tasks such as swimming and pawing during the FST (152). It is possible that exposure to DD during development has a major impact on the cortex than it does during adulthood. We cannot dismiss the fact that our animals were provided with a flotation aid to allow more confident motor behavioral scoring and guarantee that the escape deficit was primarily caused by DD treatment and not by the unnatural FST stressor.

Animals that are regularly used in research are born and grown, over many generations, under a regular 12:12 LD cycle with light intensities that differ from wild conditions. This difference in “lighting background” could predict the fact that the circadian system in these animals would evolve differently. It has become clear that unlike animals in the wild, laboratory rodents respond negatively to lighting conditions present in natural conditions (177). Constant darkness would be an unnatural condition for diurnal and nocturnal inbred laboratory rodents. Indeed, constant light deprivation became a very useful tool for studying the mechanisms involved in the etiology of depression and the identification of new and efficient therapeutic strategies. Furthermore, light-deprived animals are likely to represent a useful model for gaining new insights on the specific mechanisms which occur during brain/neuronal injury, such as neurodegeneration and inflammation and where the cell damage is induced spontaneously and not by impact/acceleration brain injury (178) or exposure to unnatural stressors (179, 180).

In short, like other aberrant lighting conditions (constant light, short day light) (14), light-deprived animals show anatomical, physiological and behavioral depression-related alterations, which are considered as clinical benchmarks of depression. In laboratory animals, light deprivation shapes the brain in developing pups and in adults, which end up with damaged neural systems, maladaptive behaviors, dysfunctional physiology, and impaired cognition, all of which are associated with oxidative damage. The detrimental effects of DD call for caution in the interpretation of experimental results and a re-evaluation of the use of “*free-running*” conditions. Such damage is progressive and the temporal evolution of its negative effects suggests that there is not a safe window for long-term experimental use.

There are still several key questions at stake. Is there a light-dependent process responsible for the neuroprotective/trophic effect? Is this process related to the well-known direct masking effect of light? Is the poor visual sensory environment a cause of depression? There would be one way to address these critical doubts. A non-photic conditioned stimulus (air flow) that was previously associated to an unconditioned stimulus (a light pulse) has the ability, by itself, to induce Fos expression in the SCN and phase shift the spontaneous activity and body temperature SCN-dependent rhythms during the subjective night, thus mimicking the effect of a pulse of light (181). This would be an alternative to keep the rhythms of light-deprived animals synchronized with the natural LD cycle. If the use of Pavlovian conditioning provides healthier light-deprived animals, then the neuroprotective/trophic effect of light *per se*, and the reduced sensory environment, could be discarded as principal factors for the genesis of the morphological, behavioral and physiological changes described previously; the synchronization of rhythms by light, known as the “masking effect of light” would be the key to this process.

## Dim red light is not an alternative

Certain housing facilities use red-tinted observation windows or hold animals under red light throughout the night to facilitate maintenance and experimental procedures. Like white light, exposure to nightly red light (DRL_N_) acts as a *zeitgeber* by altering the entrainment of circadian activity rhythms and suppresses melatonin (100). As mentioned earlier, DRL_N_ (5 lux) significantly increased locomotor activity in holding cages during the dark phase compared to a control group without any light exposure during the night (43). At higher intensities (8 lux), DRL_N_ dramatically disrupted the rhythmic circadian pattern and altered the physiological parameters of nocturnal albino rats (182). Even though there was no significant difference in the phase and period of the circadian rhythm associated with melatonin, the amplitude was reduced by a suppression of peak-dark phase secretion to almost daytime values. Moreover, the daily rhythms of arterial plasma glucose and lactate concentrations, along with arterial pO_2_ and pCO_2_, were almost identical to controls, although their levels were higher over the 24-h day. Moreover, corticosterone and insulin showed a disrupted circadian rhythm; peaks of plasma corticosterone and insulin were both phase-advanced, and the 24 h-day concentrations were reduced. In terms of leptin, 24 h-cycling was absent, the plasma peak was phase-delayed and the 24 h-day concentration was increased. Furthermore, the plasma levels of total fatty acid were elevated throughout the 24-h day and the circadian rhythm was abrogated [(182). Even at very low intensities (<1 lux), short-term (2 h) DRL_N_ exposure entrains wheel-running activity and the time of ovulation in light-deprived animals (*free-running* animals). This occurs by phase advancement when applied at the end of their active period, and by phase delay when applied at the beginning of their active period (98, 183).

Constant dim red light (DimRR) is far from an ideal option instead of DD because it also promotes circadian dysfunction. In adult nocturnal rats, DimRR, at very low intensities (0.5–1 lux), progressively reduces the amplitude of sleep-wake by decreasing the amount of sleep during the rest phase. This changes the rhythmic pattern of locomotion and drinking activities, along with brain and intraperitoneal temperature, as well as increasing the number of REM sleep episodes during the active phase (184). Long-term DimRR at higher intensity (5 lux) generates free-running locomotor activity (185, 186) and uncouples melatonin rhythm from that of locomotor activity and body temperature (185). Moreover, at 5 lux, DimRR slightly reduced the amplitude of the rhythm of *Per1* mRNA expression in the SCN and noticeably increased the overall variability of *Per1* mRNA expression in the SCN, the clock-controlled gene arylalkylamine N-acetyltransferase (*Aa-nat*) mRNA in the retina and pineal gland (186), and the plasma levels of melatonin, although their circadian rhythm persisted (185, 186). These effects of DimRR on the circadian system would result in the loss of coupling between the SCN-oscillators, thus affecting the temporal pattern and synchronization of behavioral and physiological rhythms (184–186).

In short, we must not underestimate the effect of red light, which effectively disrupts physiological and behavioral circadian rhythms. Consequently, red light, even at very low intensities, should not be regarded as functionally equivalent to darkness. For scientists who need to carry out tasks under darkness, it is extremely important to maintain light intensity at 0 lux for all wavelengths when measured at the animal's eye level.

## Concluding remarks

An increasing body of literature describes the immediate impact of light in the development of fetal rhythms and on the wellbeing of offspring and adults. Collectively, this literature aims to improve awareness in the scientific community with regard to the use of aberrant lighting conditions as a risk factor. Without light-dark transitions to reset the principal clock, the risk for lifelong physiological and behavioral maladaptive responses is increased. Dim light at night, as well as DD, can profoundly alter the circadian timing system along with the downstream control it exerts on mood, behavior, DNA repair, apoptosis, synaptic plasticity, cellular proliferation, endocrine and metabolic function, the immune system, and cognition. We cannot underestimate a direct influence of these lighting conditions on mechanisms that are independent of the circadian system, but further research is needed to understand how both direct and indirect pathways interplay and contribute to the disruptive effects of dim light at night and DD on health and wellbeing.

It is important to ensure that we control lighting in a stricter manner and to give broader consideration to the potential effects of light leakage at night. Animal facilities that do not comply with the *Guide for the Care and Use of Laboratory Animals* must be redesigned in order to improve blackout conditions. Even minimal “accidental” light exposure during the dark phase should not be disregarded, independently of the intensity and wavelength. Furthermore, a complete report, which describes lighting conditions from prenatal and postnatal periods, including the intensity and spectrum of light, should be communicated by providers to the customer. It is important that suppliers provide us with a detailed history of the lighting conditions in which our laboratory animals were reared in order to verify that such animals are appropriate for research.

In addition, the color and intensity of light at the animal's eye level need to be specified and published in detailed scientific protocols to avoid substantial variations in quality and thus increase our chances of obtaining reliable and reproducible results.

Constant darkness, which is used by chronobiologists to release an individual's rhythms of the main zeitgeber (the light), should not be considered a natural environment but rather, as a bad scenario for the wellbeing and health of nocturnal and diurnal laboratory animals. Indeed, light deprivation became an inexpensive and simple method with which to produce an anatomical, physiological and behavioral depressive phenotype in laboratory animals. Long-term light deprivation can exacerbate oxidative damage by disrupting the expression of clock genes and weakening the molecular substrates required for maintaining neuronal and synaptic function. Therefore, light should not be dismissed as an environmental condition when research studies are evaluating normal function in the whole body of laboratory animals.

In conclusion, a stable 24 h LD photoperiod with clear contrast between day and night, with safe light during the day and null light at night, is key to maintaining the circadian integrity of mammals from pre- and post-natal periods to adulthood, which in turn, will result in normal development and good health. Outside this criterion, the stability of biological function becomes altered and thus affects the wellbeing of animals, and experimental results in the short and long-term. Because of these reasons, experimental lighting conditions deserve special attention and should be periodically checked throughout the entire life of laboratory animals to provide an optimal effect and ensure good welfare. We need to reach a consensus on this matter so that we can adopt international standard criteria for photoperiods to be used for laboratory animals, which follow similar methodologies to measure the properties of light. Furthermore, the Institutional Animal Care and Use Committees should call for compliance with a universal set of rules and clearly explain how the failure to comply with such codes of practice could have very serious consequences for the animals.

## Author contributions

The author confirms being the sole contributor of this work and approved it for publication.

### Conflict of interest statement

The author declares that the research was conducted in the absence of any commercial or financial relationships that could be construed as a potential conflict of interest.

## Footnotes

^1^ARRP Guideline 22: Guidelines for the Housing of Mice in Scientific Institutions Animal Welfare Unit, NSW Department of Primary Industries, Australia.

^2^European convention for the protection of vertebrate animals used for experimental and other scientific purposes – Appendix A, Council of Europe Convention ETS 123.

## Abbreviations

DD: long-term constant darkness
DMH: dorsomedial hypothalamus
SPV: subparaventricular zone
DWL_N_: continuous dim white light at night
DBL_N_: continuous dim blue light at night
DRL_N_: continuous dim red light at night
DimRR: constant dim red light
LD: light-dark
mRGCs: melanopsin-containing ganglion cells
NA-LC: noradrenergic locus coeruleus
ROS: reactive oxygen species
SCN: suprachiasmatic nucleus.
